# Molecular imaging identifies age-related attenuation of acetylcholine in retrosplenial cortex in response to acetylcholinesterase inhibition

**DOI:** 10.1038/s41386-019-0397-5

**Published:** 2019-04-22

**Authors:** Theodosia Vallianatou, Mohammadreza Shariatgorji, Anna Nilsson, Elva Fridjonsdottir, Patrik Källback, Nicoletta Schintu, Per Svenningsson, Per E. Andrén

**Affiliations:** 1 Department of Pharmaceutical Biosciences, Medical Mass Spectrometry Imaging, National Resource for Mass Spectrometry Imaging, Science for Life Laboratory, Uppsala University, Box 591, Uppsala, SE-75124 Sweden; 2 Department of Neurology and Clinical Neuroscience, Karolinska Institutet, Stockholm, SE-17176 Sweden

**Keywords:** Biological techniques, Nervous system, Risk factors, Neurochemistry, Predictive markers

## Abstract

The neurotransmitter of the cholinergic system, acetylcholine plays a major role in the brain’s cognitive function and is involved in neurodegenerative disorders. Here, we present age-related alterations of acetylcholine levels after administration of the acetylcholinesterase inhibitor drug tacrine in normal mice. Using a quantitative, robust and molecular-specific mass spectrometry imaging method we found that tacrine administration significantly raised acetylcholine levels in most areas of sectioned mice brains, *inter alia* the striatum, hippocampus and cortical areas. However, acetylcholine levels in retrosplenial cortex were significantly lower in 14-month-old than in 12-week-old animals following its administration, indicating that normal aging affects the cholinergic system’s responsivity. This small brain region is interconnected with an array of brain networks and is involved in numerous cognitive tasks. Simultaneous visualization of distributions of tacrine and its hydroxylated metabolites in the brain revealed a significant decrease in levels of the metabolites in the 14-month-old mice. The results highlight strengths of the imaging technique to simultaneously investigate multiple molecular species and the drug–target effects in specific regions of the brain. The proposed approach has high potential in studies of neuropathological conditions and responses to neuroactive treatments.

## Introduction

Acetylcholine (ACh), the neurotransmitter of the cholinergic system, plays crucial roles in both the central (CNS) and autonomous nervous system. *Inter alia*, it is essential for healthy brain cognitive function, and one of the neurotransmitters with the greatest effects on memory [1]. Cholinergic transmission through the hippocampal formation and medial prefrontal cortex is particularly vital for cognitive function [2]. Loss of cholinergic neurons in these brain structures is associated with development of pathophysiological neurodegenerative states such as dementias, including Alzheimer’s disease (AD) [3]. This cholinergic decline is associated with aging and various other pathophysiological, genetic and environmental risk factors [4]. The effect of aging on the cholinergic system has been studied using several approaches, mainly focusing on activities of enzymes involved in ACh synthesis (choline acetyltransferase) and degradation (acetylcholinesterase, AChE) [4]. However, conflicting results have been reported [5], indicating that senescence-induced neurochemical alterations are complex.

Investigation of the presence and distribution of ACh in the CNS cholinergic pathways is challenging. ACh is very unstable postmortem, and attempts to probe the pathways’ activities using its precursor, choline, have been confounded by rapid increases in choline levels in postmortem tissue [6]. AChE is commonly used as a marker for ACh. However, it is located in both ACh neurons and cell membranes of neurons that receive cholinergic input, but are not cholinergic [7]. A further complication is that there is more than one ACh-degrading enzyme [8]. Therefore, an imaging method that provides information on brain localizations and concentrations of ACh would be highly valuable for elucidating its involvement in cognitive functions and assessing the efficacy of drugs targeting the cholinergic system.

Here, we present a quantitative matrix-assisted laser desorption ionization mass spectrometry imaging (MALDI-MSI) approach for detecting ACh directly in brain tissue sections and demonstrate its utility for simultaneously assessing effects of tacrine, an AChE inhibitor, as well as effects of age on responses to the drug. Unlike traditional brain imaging techniques, MSI is a label-free technique that enables simultaneous detection of multiple molecular species (small molecules, lipids, peptides, and proteins) at relatively high-lateral resolution [9, 10]. MALDI-MSI has been used to detect diverse exogenous and endogenous neuroactive compounds, including CNS-active drugs and neurotransmitters [11]. The development of MSI together with a well-controlled approach for tissue preparation to minimize postmortem degradation of ACh enabled us to visualize and quantify responses of ACh in specific structures in brain tissue samples to the drug. The effects of age and the well characterized AChE inhibitor tacrine on brain levels of ACh in mice provided further insight into distributions of the neurotransmitter.

## Materials and methods

### Chemicals

Chemical structures of the investigated analytes are presented in the Supplementary Material (Fig. S1) and both theoretical and empirically determined mass-to-charge (m/z) values of the molecular species used in the study are provided in Table S1. The AChE inhibitor tacrine and 9-aminoacridine (9AA) were purchased from Sigma-Aldrich (Stockholm, Sweden), while the deuterated analogues ACh-*d*_9_, ACh-*d*_4_ and CHCA-*d*_4_ were obtained from CDN isotopes (Essex, UK), BOC Sciences (NY, USA), and Ubichem (Budapest, Hungary), respectively. The solvents water, methanol and acetonitrile were of HPLC grade (VWR, Stockholm, Sweden). Trifluoroacetic acid (TFA) and 2,5-dihydroxybenzoic acid (DHB) were purchased from Merck (Darmstadt, Germany).

### Animal experiment

Mice (male C57BL/6J), aged 12 weeks (12-w, *n* = 8) and 14 months (14-m, *n* = 8) obtained from Janvier labs (Scand-las Turku, Finland) were housed under controlled temperature and humidity (20 °C, 53% humidity), as well as *ad libitum* feeding conditions, in a 12 h light/dark controlled cycle. All experiments were carried out in accordance with European Council Directive 86/609/EEC and approved by the local Animal Ethical Committee (approval Nos. N40/13 and N275-15). All efforts were made to minimize the number of animals used and their suffering. Tacrine, dissolved in saline, was administered intraperitoneally (i.p.) at a dose of 10 mg/kg to both 12-w and 14-m mice. Control 12-w and 14-m animals were injected with an equivalent amount of vehicle. All animals were euthanized by decapitation 30 min after the injection. Brains were then rapidly dissected out, snap-frozen in dry-ice cooled isopentane, and stored at −80 °C.

### Tissue processing

Tissue sectioning was performed at −20 °C using a CM1900 UV cryostat-microtome (Leica Microsystems, Welzlar, Germany). Coronal (brain levels of bregma 0.98, 0.02, and −1.06 mm) and sagittal (lateral 1.5–2.0 mm) brain tissue sections [12], were cut at a thickness of 12 μm and subsequently thaw-mounted on conductive indium tin oxide-coated glass slides (Bruker Daltonics, Bremen, Germany). The slides were stored at −80 °C. Sections were desiccated at room temperature for 15 min prior to spotting calibration standards, after which they were imaged optically using a photo scanner (Epson Perfection V500).

Sagittal brain tissue sections (*n* = 3) were used for relative determination of the brain distributions of ACh and tacrine. ACh-*d*_9_ was used as the internal standard for the MALDI-MSI analysis of ACh. Therefore, before the matrix application, a solution of ACh-*d*_9_ (0.367 μΜ) in 50% acetonitrile and 0.2% TFA was applied with an automatic TM-sprayer (HTX-Technologies LLC, Chapel Hill, NC, USA) at 90 °C, using 6 passes with a solvent flow rate of 70 μL/min, spray head velocity of 1100 mm/min, and track spacing of 2.0 mm. Addition of TFA in the solution of the internal standard prevented the enzymatic degradation of ACh. The same method was used to apply a solution of 9AA onto tissue sections (1.464 μM in 50% acetonitrile, 0.2% TFA), which served as the internal standard for the MALDI-MSI analyses of tacrine and its hydroxylated metabolites.

After experimental optimization, CHCA-*d*_4_ was used as the matrix for the MSI analysis of ACh brain levels and distributions. However, DHB provided significantly higher ionization efficiencies for tacrine and its hydroxylated metabolites. Hence, two matrix application protocols were followed. CHCA-*d*_4_, 5 mg/mL dissolved in 50% acetonitrile and 0.2% TFA, was applied using the same method as for the internal standard application. DHB (35 mg/mL in 50% acetonitrile/water and 0.2% TFA) was sprayed using eight passes with a solvent flow rate of 70 μL/min, spray head velocity of 1100 mm/min, and track spacing of 3.0 mm.

ACh levels in brain tissue were quantified using coronal brain tissue sections (*n* = 4). For this purpose, six standard solutions of ACh-*d*_4_ (2, 1.5, 1, 0.5, 0.25, and 0.125 μΜ) were prepared in 50% methanol, 0.2% TFA. The calibration standards, together with a blank (50% methanol, 0.2% TFA), were spotted on control (12-w and 14-m) mouse brain sections using a CHIP-1000 chemical inkjet printer (Shimadzu Corporation, Tokyo, Japan) with a volume of 50 nL per spot. Spots for the standard calibration were preferably applied to the cortical area, to keep tissue matrix suppression effects as constant as possible and used to determine ACh brain concentrations in control and tacrine-dosed animals of both ages. The internal standard and matrix solution (CHCA-*d*_4_) were subsequently applied as previously described. The concentration of tacrine was also measured in coronal brain tissue sections (*n* = 4) using a series of five standard solutions (5.00, 2.5, 1.25, 0.625, and 0.312 μM, 50% methanol), manually spotted on control brain tissue at a volume of 0.1 μL per spot.

### MALDI-MS imaging

All MALDI-MSI experiments were performed using a Solarix XR 7T-2ω MALDI-Fourier transform ion cyclotron resonance (FTICR) (Bruker Daltonics) mass spectrometer in positive ionization mode with a Smartbeam II 2 kHz Nd:YAG laser. The instrument was tuned for small molecules (*m/z* 107–1000), setting the time-of-flight (TOF) value at 0.500 ms and frequency at 6 MHz. After optimization, the small laser was used for the chosen lateral resolution (60–100 μm). The laser power was optimized at the start of each analysis then held constant during the MALDI-MS imaging experiment. Spectra were collected by summing signals from 100 laser shots per pixel, using red phosphorus at the appropriate mass range for calibration. The [M]^+^ ion of ACh-*d*_9_ (*m/z* 155.174046) and the [M+H]^+^ ion of 9AA (*m/z* 195.091675) were used as lock masses for MALDI-MSI of ACh and tacrine/OH-tacrine, respectively. In the case of MALDI-MSI and quantitation of ACh, continuous accumulation of selected ion (CASI) was used to improve the limit of detection toward the analyte. The quadrupole isolation *m/z* value (Q1 mass) was set at 150 *m/z* and a mass window of 40 Da was selected to include the endogenous ACh and deuterated analogues. The TOF and frequency values were adjusted to 0.450 ms and 4 MHz, respectively, while the other parameters remained the same. The CASI method was also applied for MALDI-MSI quantitation of tacrine by adjusting the parameters as follows: Q1 mass 199.0 *m/z*, mass window 50 Da, TOF 0.550 ms and frequency 6 MHz. Tissue sections were analyzed in random order to prevent possible bias due to factors such as matrix degradation or variation in mass spectrometer sensitivity. On tissue MALDI-MS/MS fragmentation experiments were performed by isolating the precursor in a 18 Da window, allowing the target ions to be selected in the quadrupole and accumulated in the collision cell. The collision energy was 35.0 V. Following MALDI-MSI analysis, the sections were histologically analyzed using Nissl staining.

### Imaging data, quantification, and statistical analysis

MSI signals from the sections were visualized using FlexImaging (v. 5.0, Bruker Daltonics). The initial MSI analysis of the sagittal brain sections was performed using SCiLS Lab (v. 2019a Pro, Bruker Daltonics). Levels of ACh, tacrine and the hydroxylated tacrine metabolites were normalized with respect to the corresponding internal standards while root-mean square normalization was applied to the rest of the data. The *m*/*z* values were extracted with a mass window of 0.3 mDa. For each analyte, the ion intensity was extracted from the mean spectrum of a specific region-of-interest (either the whole-brain tissue section or a particular brain structure) using the SCiLS Lab software. The ion intensity values were used for data exploration and statistical analysis. ACh and tacrine in brain tissue were quantified using msIQuant software [13] after conversion of the raw data into imzML format. Brain structures were annotated using a mouse brain atlas [12]. Standard curves of ACh-*d*_4_ were used for quantification of ACh, accounting for the density (1.027 g/cm^3^) of mouse brain tissue [14], the tissue section thickness (12 μm, as set in the cryomicrotome) and area (mm^2^) of deposited spots.

SPSS statistical software (v. 25.0, IBM, Armonk, NY, USA) was used for statistical analyses. Normality (Shapiro–Wilk) and homogeneity (Levene’s) tests were first applied and the ion intensity values were log-transformed to meet their requirements. Principal component analysis (PCA) and multiple linear regression were performed treating age (in weeks) as a continuous variable. Two-way ANOVA with significance level (*α*) set at 0.05 was applied to assess effects of tacrine administration and age, expressed as binary (2-level) factors on ACh levels.

## Results

### Aging and AChE inhibitor effects on ACh levels and distributions

**Fig. 1:**
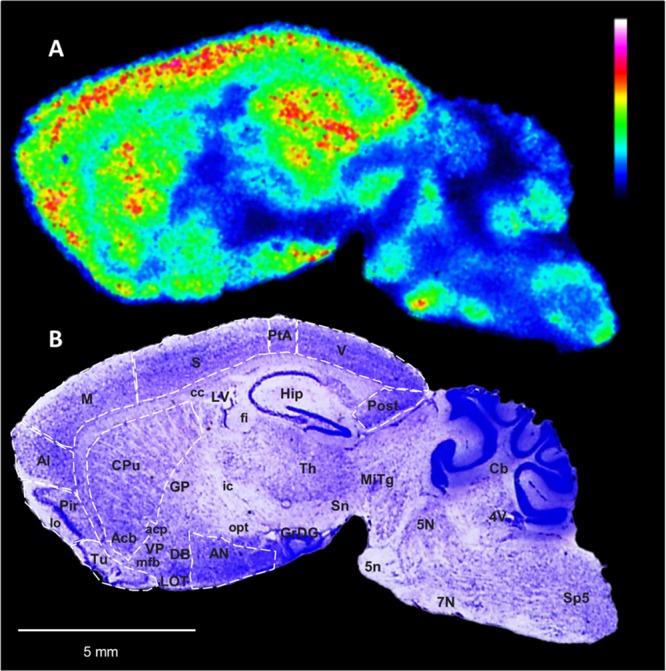
Brain distribution of ACh in a tacrine-administered 14-m mouse sagittal brain tissue section. **a** MALDI-MSI of ACh (*m/z* 146.1176, scaled to 60% of max intensity and normalized to the internal standard) in a representative sagittal mouse brain section (lateral 1.7 mm) of a 14-m tacrine-administered animal at a lateral resolution of 60 μm. **b** The tissue section was subsequently washed and subjected to Nissl staining; brain structures of interest are annotated. ACh was highly localized in the Cx and Hip, which are areas receiving cholinergic innervation from the basal forebrain cholinergic nuclei. High levels of ACh were also detected in the striatum, i.e., CPu and Acb, in which cholinergic interneurons are present, as well as in the Tu. ACh abundance was also high in the AN and Th, receiving cholinergic input from the basal forebrain and upper brainstem, respectively, and the basal forebrain nuclei (DB), which are characterized by projecting cholinergic neurons, and in certain areas of the hindbrain and cerebellum, projected mainly by the mesencephalic cholinergic neurons. Abbreviations for gray matter areas: 5N motor trigeminal nucleus, 7N facial nucleus, Acb nucleus accumbens, AI agranular insular cortex, AN amygdalar nuclei, Cb cerebellum, CPu caudate putamen, Cx cerebral cortex, DB nucleus of diagonal band, GP globus pallidus, GrDG granular cell layer of dentate gyrus, Hip hippocampus, Hyp hypothalamus, LOT nucleus of the lateral olfactory tract, M motor cortex, Mitg microcellular tegmental nucleus, Pir piriform cortex, Post postsubiculum, PtA parietal association cortex, S somatosensory cortex, Sn substantia nigra, Sp5 spinal trigeminal nucleus, Th thalamus, Tu olfactory tubercle, V visual cortex, VP ventral pallidum. Abbreviations for white matter areas: 5n trigeminal (cranial) nerve, acp anterior commissure post limb, cc corpus callosum, fi fibria of hippocampus, ic internal capsule, lo lateral olfactory tract, mfb. medial forebrain bundle, opt optic tract. Abbreviations for ventricular areas: LV lateral ventricles 4V fourth ventricle

**Fig. 2:**
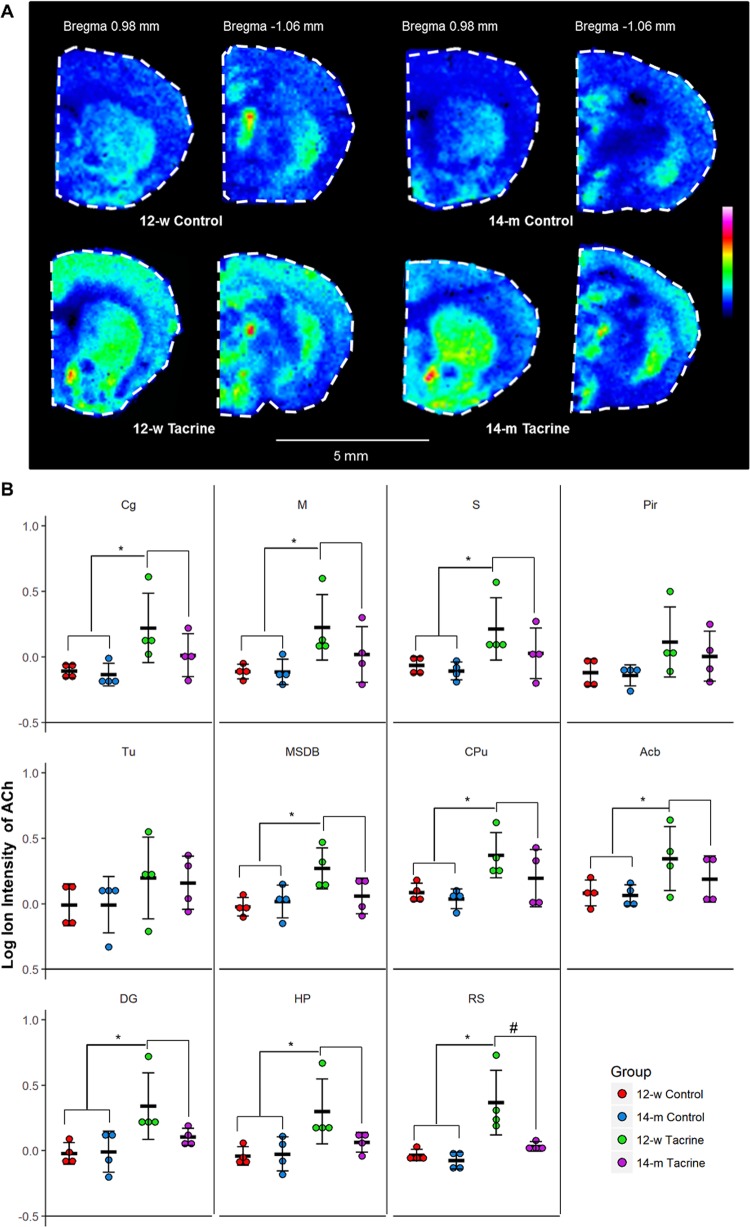
Molecule-specific imaging of ACh in coronal brain tissue sections. **a** MALDI-MSI of ACh in representative coronal brain sections of two brain levels (bregma 0.98 and −1.06 mm) [12] from 12-w to 14-m controls and tacrine-administered animals (images scaled to 40% of max intensity). **b** Dot plot of the log ion intensities of ACh in the investigated brain structures (*n* = 4). Error bars show 95% confidence interval. Significant difference (*α* = 0.05) between the two treatment groups is illustrated with (*). Significant difference (*α* = 0.05) between the 12-w tacrine and the 14-m tacrine groups in the area of the retrosplenial cortex is illustrated with (#). Abbreviations: Cg cingulate cortex, M motor cortex, S sensor cortex, Pir piriform, Tu olfactory tubercle, MSD medial septum/diagonal band, CPu caudate putamen, Acb nucleus accumbens, DG dentate gyrus, HP hippocampal proper, RS retrosplenial cortex

Sagittal mouse brain tissue sections were investigated from 12-w to 14-m control and tacrine-administered animals and the ACh distribution was consistent with previous findings [15], for example its abundance in the cortex, striatum and hippocampus, as well as in the cholinergic nuclei of the basal forebrain (Fig. 1). Quantitative imaging of levels in coronal brain tissue sections of mice of both ages (Fig. 2) confirmed previous findings regarding ACh levels in sagittal brain sections (Fig. S2) and offered detailed insights into its distribution in specific brain structures (Fig. S3).

Effects of tacrine administration and aging on ACh in cholinergic innervation structures in coronal mouse brain sections are shown in Figs. 2 and S4. A two-component PCA model constructed to explore relations between ACh levels and these factors explained 83.6% of their total variance. Close strong proximity in the resulting loadings plot (Fig. S5) indicates that whole-brain levels of tacrine were positively correlated with ACh levels in the explored brain areas. In contrast, age was negatively correlated with ACh levels as they are located in opposite quadrants of the loadings plot. This is corroborated by Pearson correlation coefficients (Table S2), which indicate a significant, positive relationship between tacrine and ACh brain levels (*r*_mean_ = 0.57) and a weakly negative correlation between age and total ACh levels (*r*_mean_ = −0.26).

**Fig. 3:**
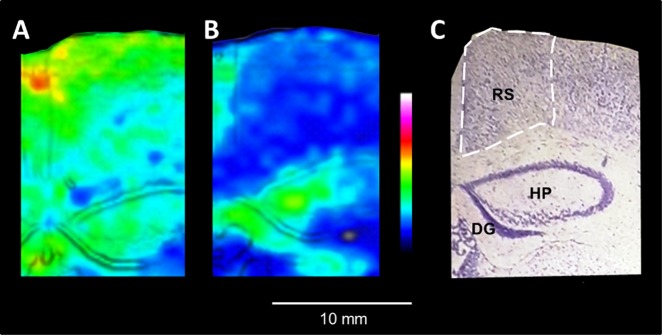
Age-related decrease of ACh concentration in retrosplenial cortex in response to acetylcholinesterase inhibition. **a**, **b** MALDI-MSI of ACh distribution in the retrosplenial cortex of 12-w and 14-m tacrine-dosed animals (100 μm lateral resolution, images scaled to 40% of max intensity). The retrosplenial cortex is highlighted by white dashed lines. The MALDI-MS and Nissl stained images are overlaid using the image fusion function implemented in msIQuant software [13] for better delineation of the brain structures. **c** The tissue section was washed and subjected to Nissl staining; brain structures of interest are annotated. Abbreviations: DG dentate gyrus, HP hippocampal proper, RS retrosplenial cortex

The brain region where the relationships between ACh levels and the two investigated factors were strongest was the retrosplenial cortex, according to the PCA, Pearson correlation analysis, and two-way ANOVA. Tacrine administration led to a significant increase in ACh levels (*P* < 0.05) in all of the examined brain areas except the olfactory tubercle and piriform cortex of 12-w animals, while effects of aging were only significant in the retrosplenial cortex (*P* = 0.014). The interaction effect of the two investigated factors was also only significant in the retrosplenial cortex (*P* = 0.044). Most importantly, these findings were reflected in the MALDI-MS images (Fig. 2), and indicated that responsivity to the drug differed between the 12-w and 14-m mice, as the ACh increase triggered by tacrine was significantly lower in the latter (Fig. 3).

ACh concentrations were measured in the same coronal brain sections (right hemisphere). At least three calibration curves per experiment were constructed, showing good linearity (*R*^2^ > 0.94, Fig. S6), a highly significant slope (*P* < 0.0005) and insignificant intercept (*P* > 0.2). The average ACh concentrations in the investigated brain areas (somatosensory cortex, caudate putamen, and hippocampus) of control and tacrine-administered animals were 7.96 (±1.37) and 14.86 (±2.65) pmol/mg tissue, respectively (Table S3).

### Imaging of tacrine in sagittal and coronal brain tissue sections

We selected 9-aminoacridine (9AA) as the internal standard for the MSI analysis due to its structural similarity to tacrine (Fig. S1), which is reflected in similarities in their physicochemical properties (Table S4). The use of 9AA as an internal standard considerably improved the normalization and visualization of tacrine distribution in the brain (Fig. S7).

**Fig. 4:**
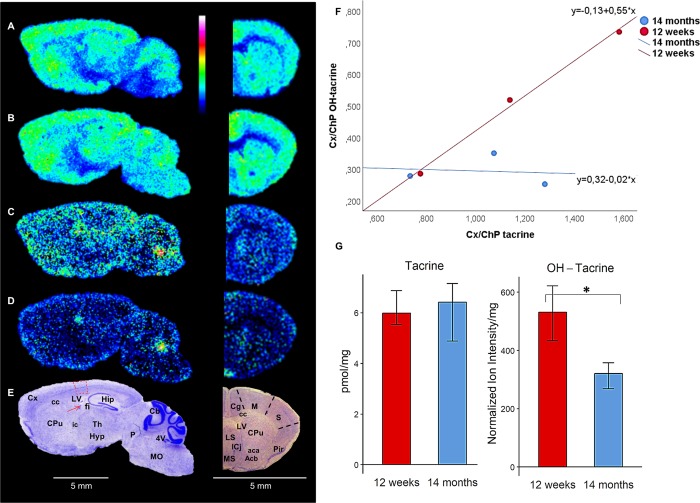
Brain distribution of tacrine and its hydroxylated metabolites after intraperitoneal administration (10 mg/kg). **a**, **b** Sagittal and coronal brain tissue sections imaged by MALDI-MS showing tacrine (*m/z* 199.123) in 12-w and 14-m mice, respectively (35% of max intensity). **c**, **d** Sagittal and coronal brain tissue sections imaged by MALDI-MS showing OH-tacrine (*m/z* 215.118) in 12-w and 14-m mice, respectively (20% of max intensity). Both analytes were imaged by MALDI-MS at a lateral resolution of 80 μm. **e** The sagittal and coronal brain tissue sections were subsequently washed and subjected to Nissl staining; brain structures of interest are annotated. Areas used for measuring the Cx/ChP ratio (ratio of a compound’s concentration in the somatosensory cortex to its concentration in the choroid plexus) in the sagittal brain tissue sections are highlighted in red. **f** Scatter plots of Cx/ChP ratios for tacrine (*X*-axis) and OH-tacrine (*Y*-axis) (*n* = 3). **g** Bar plot of the whole-brain concentration of tacrine (pmol/mg tissue) and normalized ion intensity of OH-tacrine (*n* = 4). Statistically significant differences (*P* < 0.05) are indicated with an asterisk (*). Abbreviations: Acb nucleus accumbens, Cb cerebellum, Cg cingulate cortex, ChP choroid plexus, CPu caudate putamen, Cx cortex, Hip hippocampus, Hyp hypothalamus, LS lateral septal nucleus, M motor cortex, MO medulla oblongata, MS medial septal nucleus, P pons, Pir piriform cortex, S somatocensory cortex, Th thalamus, aca anterior commissure anterior part, cc corpus callosum, f fimbria of the hippocampus, ic internal capsule, 4V fourth ventricle, LV lateral ventricle

Tacrine (*m/z* 199.123) was widely distributed throughout the brain, with similar localization in the gray matter in both 12-w and 14-m animals (Fig. 4a, b). The drug was highly localized in the cortex, especially the cingulate cortex, caudate putamen, nucleus accumbens, lateral and medial septal nucleus, hippocampus, thalamus and cerebellum, in accordance with early autoradiographic studies [16]. Imaging of the hydroxylated tacrine metabolites [17, 18] (1-OH-tacrine, 2-OH-tacrine or 4-OH-tacrine, *m/z* 215.118) in the same experiments indicated that they were more abundant in the ventricular areas (lateral and fourth ventricles) than in gray and white matter areas (Fig. 4c, d). Delineation of the brain structures was provided by corresponding stained mouse brain sections (Fig. 4e). Normalized ion intensities of tacrine and OH-tacrine extracted from a small area of the somatosensory cortex and choroid plexus of the lateral ventricle in sagittal brain sections of mice of both ages, and ratios of both compounds’ intensities in these tissues (designated Cx/ChP ratios) were calculated. It should be mentioned that pixels showing high intensity of heme B were excluded from the examined somatosensory cortex area, as they may be related to the amount of OH-tacrine that has not penetrated the blood–brain barrier (BBB) [19]. The Cx/ChP ratios were significantly higher for tacrine than for OH-tacrine (*P* < 0.01), indicating that the extent of the entrance to the brain parenchyma is considerably higher for the parent drug than for its metabolites (Fig. 4f). Concentrations of tacrine in coronal mouse brain sections of tacrine-administered 12-w and 14-m mice were found to be very similar (6.21 ± 0.34 and 6.02 ± 0.57 pmol/mg tissue, respectively) (Fig. 4g). In contrast, and most importantly, whole-brain tissue levels of OH-tacrine were found to be significantly lower in the 14-m animals (*P* < 0.05, Fig. 4g).

Both tacrine and OH-tacrine were identified by direct tandem MS (MS/MS) imaging of the tissue sections (Fig. S8). It was, however, not possible to determine the position of the metabolite’s hydroxyl group by MS/MS [17]. The product ion spectra confirmed the presence of tacrine and OH-tacrine in the brain tissue [17].

## Discussion

The presented MALDI-MSI technique provided robust indications of ACh levels and distributions in mouse brain sections, together with new insights into effects of the AChE inhibitory drug tacrine and aging. Tacrine induced substantial increases in ACh levels in most examined areas of brains of both 12-w and 14-m mice, but it induced significantly lower increases in levels of ACh (and its hydroxylated metabolite) in the retrosplenial cortex of the 14-m mice than in the younger animals.

The cholinergic system plays vital roles in the CNS and maintenance of cognitive health. Neurodegenerative conditions, such as AD and other dementias, are associated with severe decline of cholinergic neurons in certain brain structures. This is becoming increasingly important with increases in occurrence of neurodegenerative diseases, partly attributed to lifespan extensions [20]. AChE inhibitors, which increase levels of available ACh in synapses, are still prevalent medications against dementias. However, further advances are needed to improve their efficacy and/or develop new therapeutic strategies.

Investigation of the relationship between aging and cholinergic decline has been challenging [5, 21]. According to the cholinergic hypothesis of geriatric memory dysfunction [5, 22], there is a strong connection between basal forebrain cholinergic loss and cognitive impairment. However, some studies indicate that the connection between normal aging and perturbance of cholinergic inputs in the cortex and hippocampus is not entirely correct and that cholinergic dysfunction is rather associated with pathological aging [4, 21]. Moreover, it has been recently suggested that dendritic, axonal and synaptic degeneration causes cholinergic loss in normal aging [21]. The conflicting results of previous studies can be ascribed to differences in experimental species, ages of the animals, procedures and tissues selected for comparison [4]. The human brain has been found to be more susceptible to cholinergic decline by aging than animal models, but this finding can be attributed to undetected early stage AD, which is not present in the models [4]. Variations among tissues pose further complications. Aging does not reportedly affect whole-brain concentrations of choline and ACh [23], but reduces extracellular levels of ACh (measured with microdialysis and related to the neurotransmitter’s release) [24, 25]. ACh synthesis and release, especially stimulation-induced changes in rates of these processes, are also reportedly altered by aging [4]. In addition, the cholinergic system’s responsivity to cholinergic agonists is reportedly altered by normal aging [4], but again conflicting results have been obtained in some studies [4, 26].

Here, the MALDI-MS imaging showed that aged mice had less responsivity than younger animals to tacrine in the retrosplenial cortex, a substantial transit region between the hippocampus and cingulate cortex. The retrosplenial cortex, as well as the hippocampus, receives cholinergic input from the medial septal nucleus and diagonal band in the basal forebrain [27, 28]. The retrosplenial cortex and hippocampus have complementary roles in cognitive function and spatial navigation, and seem to be interrelated [27]. Most importantly, the retrosplenial cortex is reportedly one of the first brain regions to display pathological effects in early onset AD and mild cognitive impairment [29, 30]. Atrophy of the retrosplenial cortex has also been associated with incipient AD [31]. Whether or not it is more vulnerable to degeneration than the hippocampus has been debated [31], but no significant correlation has been observed between the degree of atrophy in the hippocampus and retrosplenial cortex in AD, suggesting that onset of degeneration differs in these two brain areas [32].

MALDI-MSI is a technique that can provide multimolecular information and has been previously used to investigate the brain distribution of ACh by various authors, including us [11, 33–35]. However, this study is the first to simultaneously image a drug and effects on its target, and it provides further insights into the specific localization of the neurotransmitter, as well as alterations triggered by the drug and aging. The wide distribution of tacrine in the brain, as delineated by the MSI data, is indicative of high BBB transport of the compound, as previously reported [36, 37]. In contrast to tacrine, higher levels of OH-tacrine were detected in the choroid plexus than in the brain parenchyma, indicating lower rates of BBB transport. Tacrine has three major bioactive metabolites (1-OH-tacrine, 2-OH-tacrine, and 4-OH-tacrine), where 1-OH-tacrine and 4-OH-tacrine have been detected and quantified in the brain [17, 38]. The main brain metabolite was reported to be 1-OH-tacrine and was approximately five times higher than 4-OH-tacrine [38]. Previous studies found that brain concentrations of hydroxylated metabolites of tacrine were considerably lower than those of tacrine [18, 38], presumably due to differences in their physicochemical properties, notably lipophilicity and hydrogen bonding capacity. This is further supported by the lack of brain in vitro metabolism of tacrine to its hydroxylated derivatives [18], limiting the possibility of OH-tacrine formation in the brain.

While the whole-brain concentration of tacrine was not affected by age, the brain abundance of OH-tacrine was significantly lower in the 14-m mice than in the younger animals. Previous pharmacokinetic studies on AChE inhibitors in young and old rats have found that the brain/blood ratio of tacrine was similar at both ages [39]. However, slightly lower clearance was detected in the old animals [39], which may be related to a lower rate of OH-tacrine formation. Activities of the drug-metabolizing enzyme CYP1A2, which catalyzes transformation of tacrine to OH-tacrine, decline with aging [40], which may affect overall formation of the metabolite and, thus, its brain levels. Aging seems to increase brain permeability [41], suggesting that it should also increase brain entrance, but its concomitant effects on metabolic reduction and BBB expression of potentially involved drug influx transporters [42] may account for the lower brain levels of the metabolite observed in older animals here. 1-OH-tacrine, also known as velnacrine, has also demonstrated AChE inhibitory activity [43]. However, in the present study, effects of tacrine administration on brain levels of ACh were mainly attributed to the parent drug, as the metabolite has limited brain entrance and lower potency than tacrine (IC_50_: 1.30 and 0.23 μM, respectively) [43].

In summary, by using MALDI-MSI [44], which enables the simultaneous mapping of ACh, tacrine and its metabolites, we show that normal aging affects the responsivity of the cholinergic system in the retrosplenial cortex.

## Funding and disclosure

This work was financially supported by the Swedish Research Council (Medicine and Health, grant No. 2018–03320, Natural and Engineering Science, grant No. 2018–05501), ARIADME, a European Community’s Seventh Framework Program (FP7 ITN), under grant agreement No. 607517, the Swedish Brain Foundation, the Swedish Foundation for Strategic Research (grant No. RIF14-0078), and the Science for Life Laboratory. The authors declare no competing interests.

## Supplementary information

Molecular imaging identifies age-related attenuation of acetylcholine in retrosplenial cortex in response to acetylcholinesterase inhibition

## References

[1] Blokland A (1995). Acetylcholine: a neurotransmitter for learning and memory?. Brain Res Rev.

[2] Euston DR, Gruber AJ, McNaughton BL (2012). The role of medial prefrontal cortex in memory and decision making. Neuron.

[3] Gielow MR, Zaborszky L (2017). The input-output relationship of the cholinergic basal forebrain. Cell Rep.

[4] Decker MW (1987). The effects of aging on hippocampal and cortical projections of the forebrain cholinergic system. Brain Res Rev.

[5] Bartus RT, Dean RL, Beer B, Lippa AS (1982). The cholinergic hypothesis of geriatric memory dysfunction. Science.

[6] Weintraub ST, Modak AT, Stavinoha WB (1976). Acetylcholine: postmortem increases in rat brain regions. Brain Res.

[7] Silman I, Sussman JL (2005). Acetylcholinesterase: ‘classical’ and ‘non-classical’ functions and pharmacology. Curr Opin Pharmacol.

[8] Greig NH, Lahiri DK, Sambamurti K (2002). Butyrylcholinesterase: an important new target in Alzheimer’s disease therapy. Int Psychogeriatr.

[9] Caprioli RM, Farmer TB, Gile J (1997). Molecular imaging of biological samples: localization of peptides and proteins using MALDI-TOF MS. Anal Chem.

[10] Nilsson A, Goodwin RJ, Shariatgorji M, Vallianatou T, Webborn PJ, Andren PE (2015). Mass spectrometry imaging in drug development. Anal Chem.

[11] Shariatgorji M, Nilsson A, Goodwin RJ, Kallback P, Schintu N, Zhang X (2014). Direct targeted quantitative molecular imaging of neurotransmitters in brain tissue sections. Neuron.

[12] Paxinos G, Franklin KBJ (2013). Paxinos and Franklin’s the Mouse Brain in Stereotaxic Coordinates.

[13] Kallback P, Nilsson A, Shariatgorji M, Andren PE (2016). msIQuant—quantitation software for mass spectrometry imaging enabling fast access, visualization, and analysis of large data sets. Anal Chem.

[14] Barber TW, Brockway JA, Higgins LS (1970). The density of tissues in and about the head. Acta Neurol Scand.

[15] Cuello AC, Sofroniew MV (1984). The anatomy of the CNS cholinergic neurons. Trends Neurosci.

[16] McNally W, Roth M, Young R, Bockbrader H, Chang T (1989). Quantitative whole-body autoradiographic determination of tacrine tissue distribution in rats following intravenous or oral dose. Pharm Res.

[17] Gao HY, Deng SB, Obach RS (2010). A simple liquid chromatography-tandem mass spectrometry method to determine relative plasma exposures of drug metabolites across species for metabolite safety assessments. Drug Metab Dispos.

[18] McNally WP, Pool WF, Sinz MW, Dehart P, Ortwine DF, Huang CC (1996). Distribution of tacrine and metabolites in rat brain and plasma after single-and multiple-dose regimens—evidence for accumulation of tacrine in brain tissue. Drug Metab Dispos.

[19] Liu X, Ide JL, Norton I, Marchionni MA, Ebling MC, Wang LY (2013). Molecular imaging of drug transit through the blood-brain barrier with MALDI mass spectrometry imaging. Sci Rep.

[20] Bishop NA, Lu T, Yankner BA (2010). Neural mechanisms of ageing and cognitive decline. Nature.

[21] Schliebs R, Arendt T (2011). The cholinergic system in aging and neuronal degeneration. Behav Brain Res.

[22] Schliebs R, Arendt T (2006). The significance of the cholinergic system in the brain during aging and in Alzheimer’s disease. J Neural Transm.

[23] Gibson GE, Peterson C, Jenden DJ (1981). Brain acetylcholine synthesis declines with senescence. Science.

[24] Pepeu G, Giovannini MG (2004). Changes in acetylcholine extracellular levels during cognitive processes. Learn Mem.

[25] Scali C, Giovannini MG, Prosperi C, Bartolini L, Pepeu G (1997). Tacrine administration enhances extracellular acetylcholine in vivo and restores the cognitive impairment in aged rats. Pharmacol Res.

[26] Lamour Y, Dutar P, Jobert A (1985). Cerebral neocortical neurons in the aged rat: spontaneous activity, properties of pyramidal tract neurons and effect of acetylcholine and cholinergic drugs. Neuroscience.

[27] Anzalone S, Roland J, Vogt B, Savage L (2009). Acetylcholine efflux from retrosplenial areas and hippocampal sectors during maze exploration. Behav Brain Res.

[28] Gage SL, Keim SR, Simon JR, Low WC (1994). Cholinergic innervation of the retrosplenial cortex via the fornix pathway as determined by high affinity choline uptake, choline acetyltransferase activity, and muscarinic receptor binding in the rat. Neurochem Res.

[29] Nestor PJ, Fryer TD, Ikeda M, Hodges JR (2003). Retrosplenial cortex (BA 29/30) hypometabolism in mild cognitive impairment (prodromal Alzheimer’s disease). Eur J Neurosci.

[30] Pengas G, Williams GB, Acosta-Cabronero J, Ash TW, Hong YT, Izquierdo-Garcia D (2012). The relationship of topographical memory performance to regional neurodegeneration in Alzheimer’s disease. Front Aging Neurosci.

[31] Pengas G, Hodges JR, Watson P, Nestor PJ (2010). Focal posterior cingulate atrophy in incipient Alzheimer’s disease. Neurobiol Aging.

[32] Tan RH, Wong S, Hodges JR, Halliday GM, Hornberger M (2013). Retrosplenial cortex (BA 29) volumes in behavioral variant frontotemporal dementia and Alzheimer’s disease. Dement Geriatr Cogn Disord.

[33] Shariatgorji M, Nilsson A, Goodwin RJA, Svenningsson P, Schintu N, Banka Z (2012). Deuterated matrix-assisted laser desorption ionization matrix uncovers masked mass spectrometry imaging signals of small molecules. Anal Chem.

[34] Sugiura Y, Zaima N, Setou M, Ito S, Yao I (2012). Visualization of acetylcholine distribution in central nervous system tissue sections by tandem imaging mass spectrometry. Anal Bioanal Chem.

[35] Ye H, Wang JX, Greer T, Strupat K, Li LJ (2013). Visualizing neurotransmitters and metabolites in the central nervous system by high resolution and high accuracy mass spectrometric imaging. ACS Chem Neurosci.

[36] Summerfield SG, Zhang Y, Liu H (2016). Examining the uptake of central nervous system drugs and candidates across the blood-brain barrier. J Pharm Exp Ther.

[37] Telting-Diaz M, Lunte CE (1993). Distribution of tacrine across the blood-brain barrier in awake, freely moving rats using in vivo microdialysis sampling. Pharm Res.

[38] Qian S, Wo SK, Zuo Z (2012). Pharmacokinetics and brain dispositions of tacrine and its major bioactive monohydroxylated metabolites in rats. J Pharm Biomed Anal.

[39] Goh CW, Aw CC, Lee JH, Chen CP, Browne ER (2011). Pharmacokinetic and pharmacodynamic properties of cholinesterase inhibitors donepezil, tacrine, and galantamine in aged and young lister hooded rats. Drug Metab Dispos.

[40] Kinirons MT, O’Mahony MS (2004). Drug metabolism and ageing. Br J Clin Pharm.

[41] Marques F, Sousa JC, Sousa N, Palha JA (2013). Blood-brain-barriers in aging and in Alzheimer’s disease. Mol Neurodegener.

[42] Wu KC, Lu YH, Peng YH, Tsai TF, Kao YH, Yang HT (2015). Decreased expression of organic cation transporters, Oct1 and Oct2, in brain microvessels and its implication to MPTP-induced dopaminergic toxicity in aged mice. J Cereb Blood Flow Metab.

[43] Shutske GM, Bores GM, Bradshaw KC, Huger FP, Kapples KJ, Larsen RD (1992). Synthesis and biological-activity of putative monohydroxylated metabolites of velnacrine. Bioorg Med Chem Lett.

[44] Shariatgorji M, Svenningsson P, Andren PE (2014). Mass spectrometry imaging, an emerging technology in neuropsychopharmacology. Neuropsychopharmacology.

