# Supplementary material for: Molecular imaging identifies age-related attenuation of acetylcholine in retrosplenial cortex in response to acetylcholinesterase inhibition
Source: Neuropsychopharmacology. 2019 Apr 22;44(12):2091–8. doi: 10.1038/s41386-019-0397-5 (PMC6887892; doi:10.1038/s41386-019-0397-5)
Supplement: Supplementary file 1 — Molecular imaging identifies age-related attenuation of acetylcholine in retrosplenial cortex in response to acetylcholinesterase inhibition [file 41386_2019_397_MOESM1_ESM.pdf]

Supplementary Information

Molecular imaging identifies age-related attenuation of acetylcholine in retrosplenial cortex in response to acetylcholinesterase inhibition

Theodosia Vallianatou[1], Mohammadreza Shariatgorji[1], Anna Nilsson[1], Elva Fridjonsdottir[1], Patrik Källback[1], Nicoletta Schintu[2], Per Svenningsson[2], Per E. Andrén[1]

[1] Medical Mass Spectrometry Imaging, National Resource for Mass Spectrometry Imaging, Science for Life Laboratory, Department of Pharmaceutical Biosciences, Uppsala University, Box 591, SE-75124, Uppsala, Sweden

[2] Department of Neurology and Clinical Neuroscience, Karolinska Institutet, 17176, Stockholm, Sweden

Corresponding author. Dr. Per E. Andrén, Medical MS Imaging, Dept. of Pharmaceutical Biosciences, Uppsala University, Box 591, SE-75124, Uppsala, Sweden. Phone, +46 – 471 7206. Email, per.andren@farmbio.uu.se

Supplementary Information includes,

Supplementary Figures	S1 – S8	p. 2 – 10
Supplementary Tables	S1 – S4	p. 11 – 14
References		p. 15

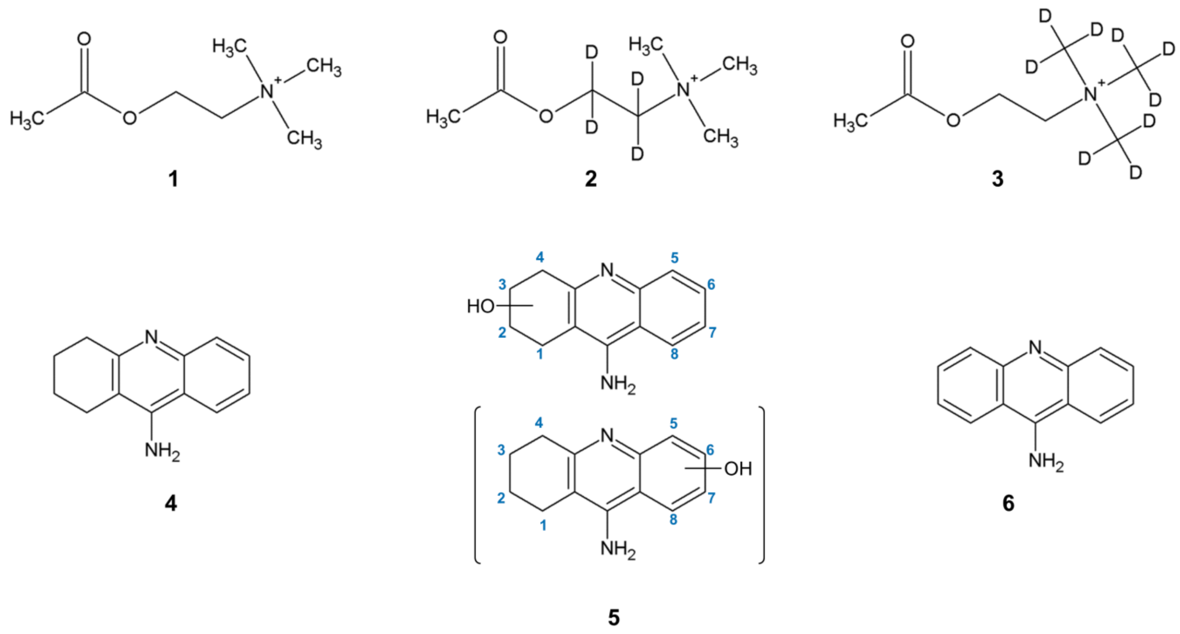

Figure S1. Chemical structures of the investigated analytes and their internal standards. 1. Acetylcholine, 2. Acetylcholine-d_4, 3. Acetylcholine-d_9, 4. Tacrine, 5. Hydroxylated metabolites of tacrine, 6. 9-aminoacridine.

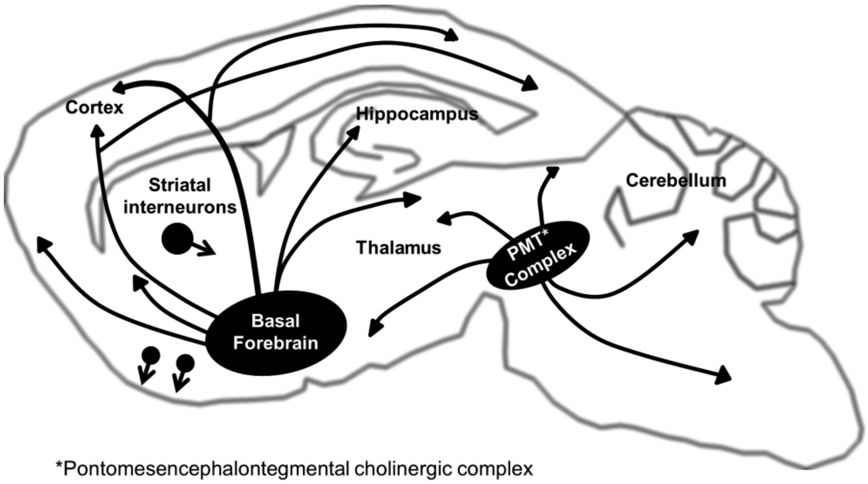

Figure S2. Schematic representation of the major cholinergic pathways in the mouse brain. These pathways have been identified by histochemical techniques for detecting acetylcholinesterase. The main source of cholinergic input to the cerebral cortex and hippocampus is the basal forebrain complex (nucleus basalis, substantia innominata and horizontal diagonal band). The pontomesencephalotegmental (PMT) cholinergic complex preferentially innervates the brain stem and midbrain targets (hindbrain, thalamus, hypothalamus and basal forebrain). Within the striatum (the caudate-putamen) are numerous cholinergic interneurons, rather than projection neurons, involved in the local circuitry.

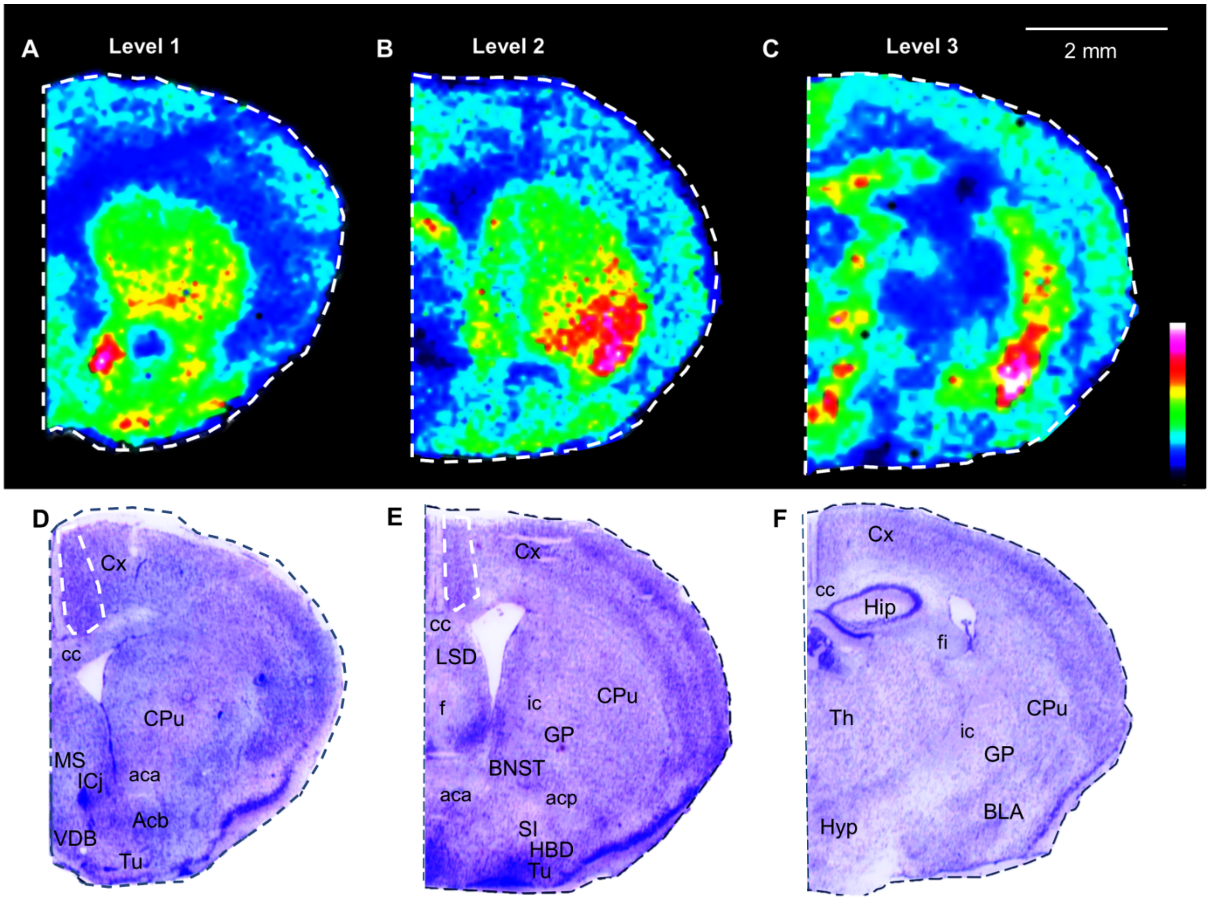

Figure S3. Distribution of ACh in coronal mouse brain tissue sections of three levels.

(A, B, C) MALDI-MSI of ACh (m/z 146.1176) in coronal mouse brain sections (right hemisphere) of a 14-month-old tacrine-administered animal at brain levels of bregma 0.98 mm (Level 1), 0.02 mm (Level 2), and -1.06 mm (Level 3) [1], respectively. Images scaled to 60% of max intensity, normalized to the internal standard, and acquired at a lateral resolution of 100 μm. (D, E, F) The tissue sections were subsequently washed and subjected to Nissl staining; brain structures of interest are annotated. The medial prefrontal cortex (mPFC) is highlighted by the white dashed line. Abbreviations for grey matter areas: Acb, nucleus accumbens; BLA, basolateral amygdalar nucleus; BNST, bed nuclei of the stria terminalis; CPu, caudate putamen; Cx, cerebral cortex; GP, globus pallidus, HDB, horizontal limb of the diagonal band; Hip,

hippocampus; Hyp, hypothalamus; ICj, islands of Calleja; LSD, lateral septal nucleus dorsal; MS, medial septal nucleus; Si, substantia innominata; Th, thalamus; Tu, olfactory tubercle; VDB, ventricular limb of the diagonal band. Abbreviations for white matter areas; aca, anterior part of anterior commissure; acp, posterior part of anterior commissure; cc, corpus callosum; f, fornix; fi, fibria of hippocampus; ic, internal capsule.

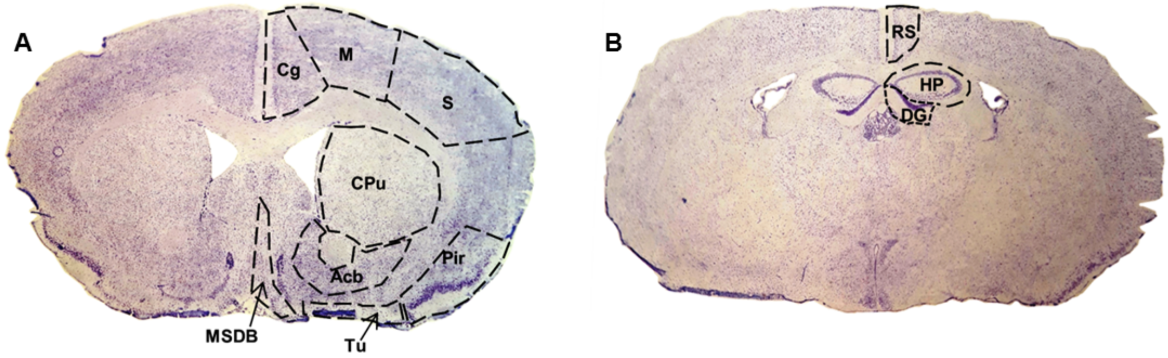

Figure S4. Illustration of the investigated brain regions in Nissl stained coronal mouse brain sections.

Abbreviations: Cg, cingulate cortex; M, motor cortex; S, sensor cortex; Pir, piriform; Tu, olfactory tubercle; MSDB, medial septum/diagonal band; CPu, caudate putamen; Acb, nucleus accumbens; RS, Retrosplenial cortex; HP, hippocampal proper; DG, dentate gyrus.

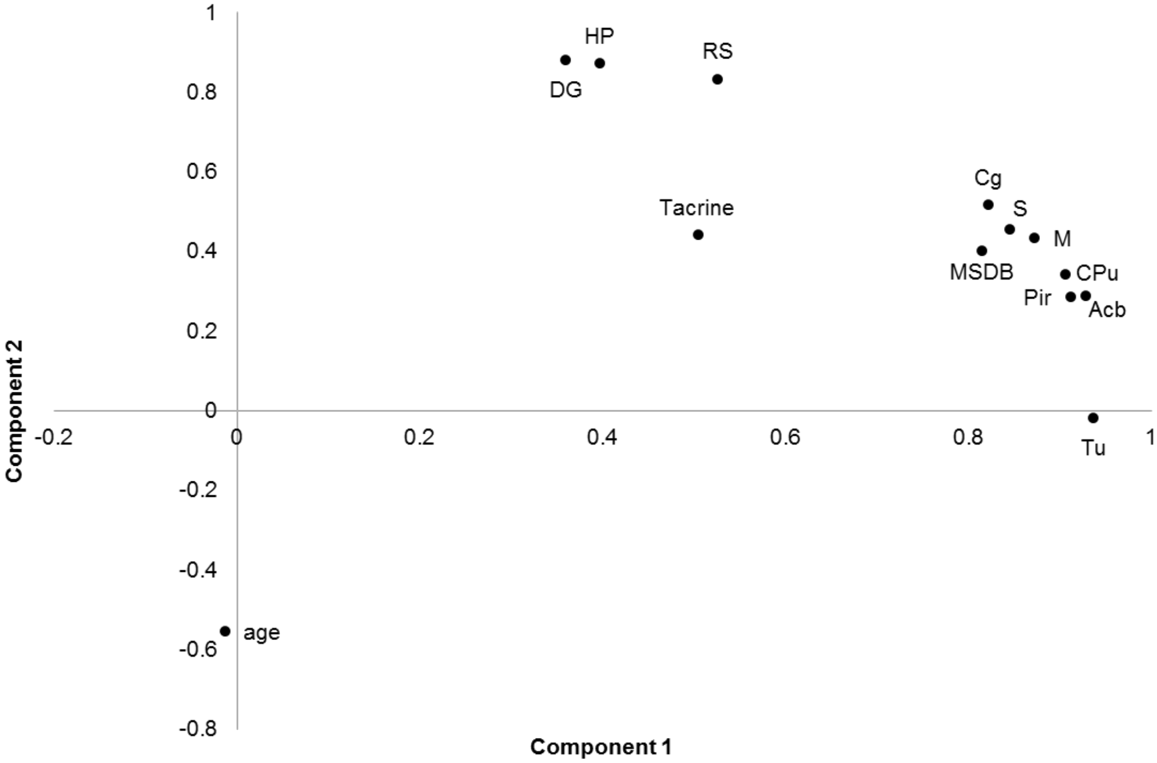

Figure S5. Loadings plot for the two-component principal component analysis (PCA) model constructed to explore associations between ACh levels in selected brain areas, tacrine administration and age (12-w and 14-m), using log ion intensities of ACh in the investigated brain areas and log ion intensities of tacrine in whole brain (n=4).

Abbreviations: Cg, cingulate cortex; M, motor cortex; S, sensor cortex; Pir, piriform; Tu, olfactory tubercle; MSDB, medial septum/diagonal band; CPu, caudate putamen; Acb, nucleus accumbens; RS, retrosplenial cortex; HP, hippocampal proper; DG, dentate gyrus.

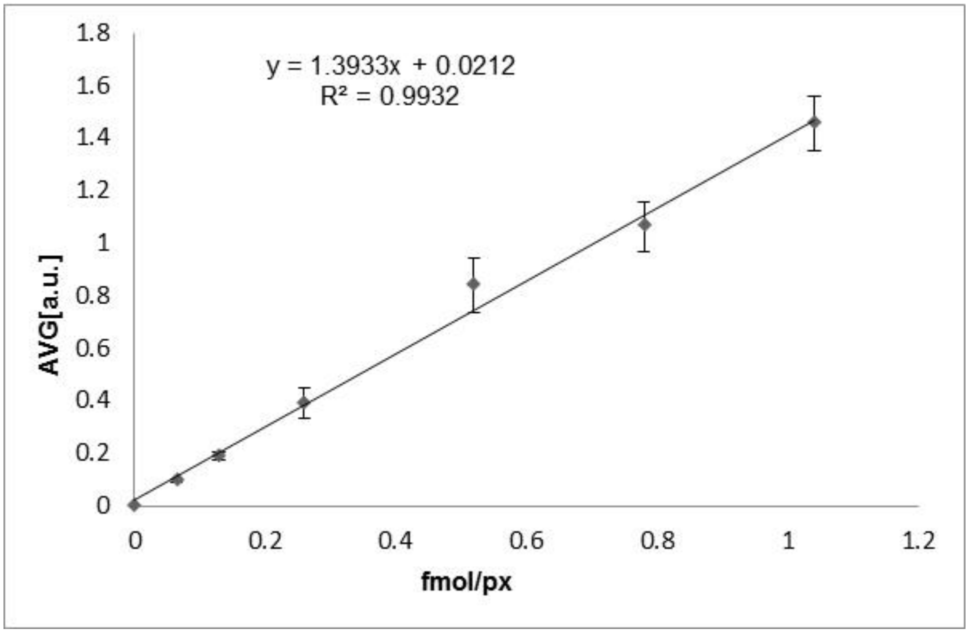

Figure S6. Calibration curve obtained by applying standards to control coronal brain sections (four replicates), demonstrating the linearity of the MSI signals.

The calibration curve was created using the msIQuant software [2], after converting the MALDI-MSI data into the appropriate format [3,4]. Brain tissue density of 1.027 g/cm^3 was considered for performing the calculations [5].

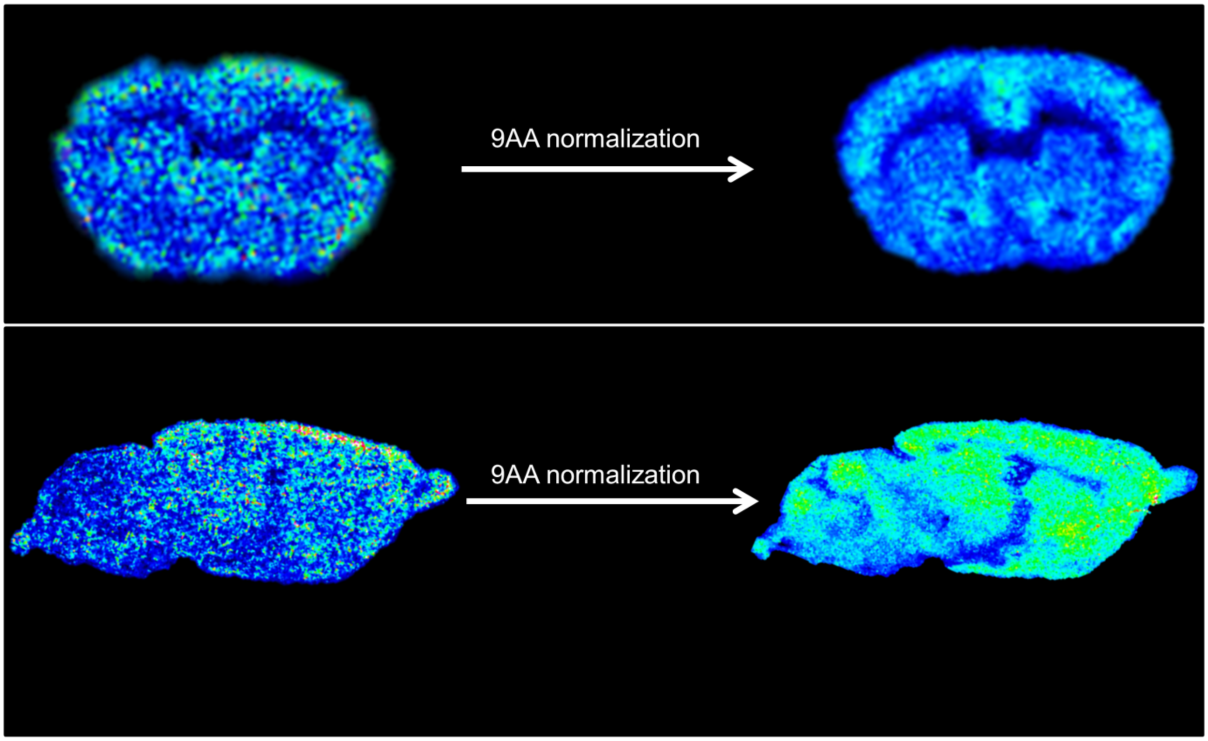

Figure S7. Illustration of improvement of the MALDI-MSI data obtained by normalizing tacrine ion intensity values using 9AA as the internal standard.

The MALDI-MS images were obtained in positive mode by using DHB as matrix. The internal standard 9AA (1.464 µM) was applied prior to the DHB matrix application.

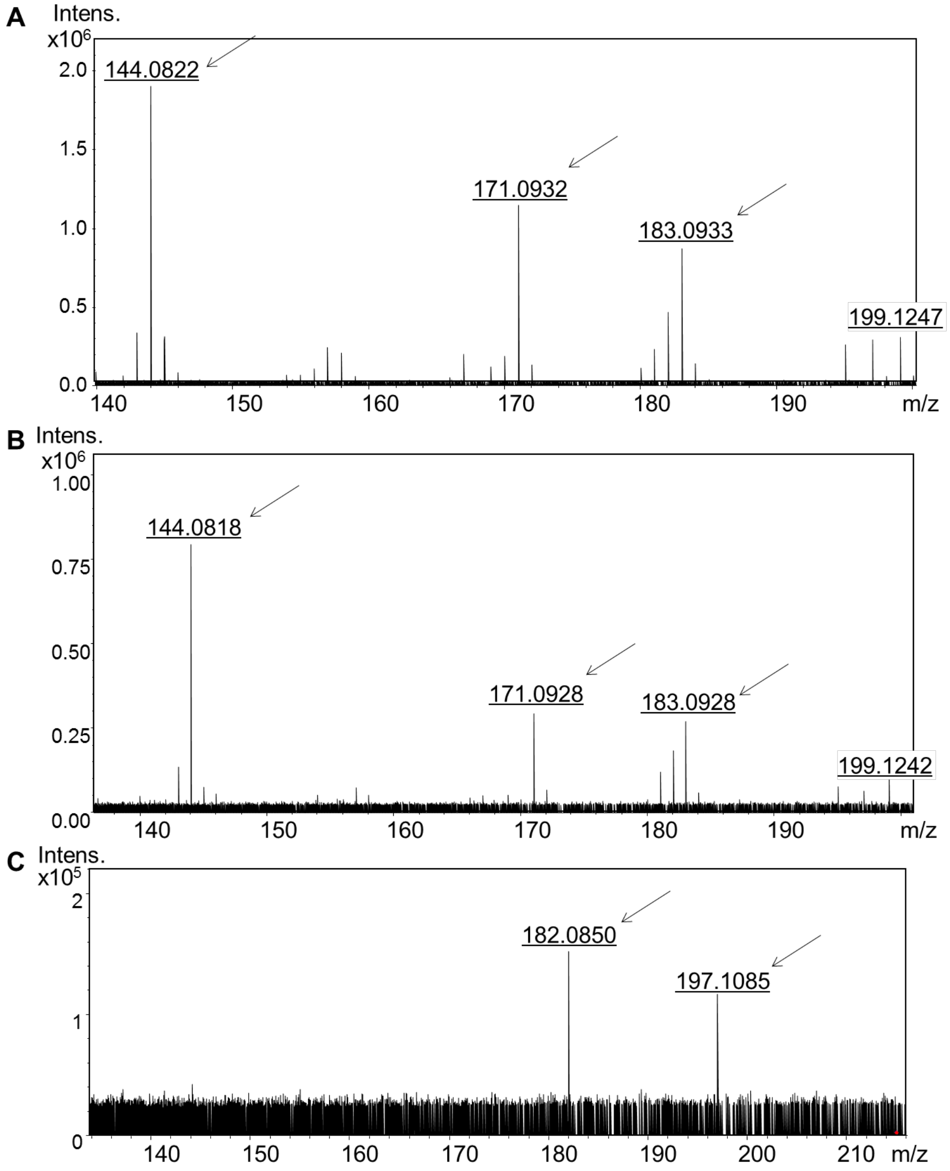

Figure S8. MALDI-MS/MS product ion mass spectra of tacrine and OH-tacrine.
(A) tacrine standard (0.1 µL of 100 µg/mL) spotted on metal MALDI target plate, (B) tacrine from the cortical area of a brain tissue sections from a tacrine-administered mouse, and (C) OH-tacrine obtained from the cortical area of a brain tissue sections from a tacrine-administered mouse. Product ions are annotated with black arrows. DHB was used as the MALDI matrix. The product ions from OH-tacrine were confirmed with a previously reported study [6].

Table S1. Molecular formula and mass accuracy of the investigated analytes and corresponding deuterated analogues.

Compound	Molecular formula	Theoretical *m/z*	Experimental *m/z*	Mass accuracy (ppm)
Acetylcholine	$C_7H_{16}NO_2$	146.117555	146.11755(±0.00003)	-0.03
Acetylcholine-*d*9	$C_7H_7D_9NO_2$	155.174046	155.17398(±0.00002)	-0.42
Acetylcholine-*d*4	$C_7H_{12}D_4NO_2$	150.142662	150.14262(±0.00004)	-0.28
Tacrine	$C_{13}H_{14}N_2$	199.122975	199.12292(±0.00012)	-0.28
OH-tacrine	$C_{13}H_{14}N_2O$	215.117890	215.11781(±0.00020)	-0.37
9-aminoacridine	$C_{13}H_{10}N_2$	195.091675	195.091583(±0.00017)	-0.47

Table S2. Pearson correlation matrix of the ACh and tacrine log ion intensities and age.

	Age	Tacrine
**Cg**	-0.29	.62*
**M**	-0.25	.60*
**S**	-0.3	.58*
**Pir**	-0.17	.54*
**Tu**	-0.04	0.43
**MSDB**	-0.23	.55*
**CPu**	-0.31	.62**
**Acb**	-0.24	.55*
**DG**	-0.28	.60*
**HP**	-0.29	.59*
**RS**	-0.45	.62*
**age**	**1**	0
**Tacrine**	0	**1**
**r_{mean}**	-0.26	0.57

* Correlation significant at the P<0.05 level (2-tailed).
** Correlation significant at the P<0.01 level (2-tailed).

Table S3. Average ACh concentration per brain area in the four investigated groups.

	Brain Area	Average ACh Concentration (pmol/mg brain tissue)
	Cortex	7.40 (±0.57)
12-w Control	Caudate putamen	11.34 (±0.94)
	Hippocampus	5.60 (±0.43)
	Cortex	7.00 (±0.75)
14-m Control	Caudate putamen	9.88 (±0.91)
	Hippocampus	6.50 (±0.62)
	Cortex	17.36 (±6.17)
12-w Tacrine	Caudate putamen	23.70 (±5.39)
	Hippocampus	13.54 (±4.48)
	Cortex	10.63 (±2.58)
14-m Tacrine	Caudate putamen	15.94 (±3.85)
	Hippocampus	8.01 (±0.77)

Table S4. Molecular and physicochemical properties of the investigated compounds.

Properties	Tacrine	OH-tacrine	9AA
Molecular weight (g/mol)	198.269	214.263	194.237
Hydrogen Bond Donors	1	2	1
Hydrogen Bond Acceptors	2	3	2
Rotatable Bonds	0	0	0
Topological Polar Surface Area ($Å^2$)	38.9	59.1	38.9
logP	2.71	1.4	2.61
pKa ($-NH_2$)	9.95	n.d.*	9.99

*n.d., not determined

References

1 Paxinos G, Franklin KBJ. Paxinos and Franklin's the mouse brain in stereotaxic coordinates. 4th ed. Elsevier Academic Press: Amsterdam; 2013.

2 Kallback P, Nilsson A, Shariatgorji M, Andren PE. msIQuant - Quantitation Software for Mass Spectrometry Imaging Enabling Fast Access, Visualization, and Analysis of Large Data Sets. Analytical Chemistry. 2016;88(8):4346-53.

3 Race AM, Styles IB, Bunch J. Inclusive sharing of mass spectrometry imaging data requires a converter for all. J Proteomics. 2012;75(16):5111-2.

4 Schramm T, Hester A, Klinkert I, Both JP, Heeren RM, Brunelle A, et al. imzML--a common data format for the flexible exchange and processing of mass spectrometry imaging data. J Proteomics. 2012;75(16):5106-10.

5 Barber TW, Brockway JA, Higgins LS. The density of tissues in and about the head. Acta Neurol Scand. 1970;46(1):85-92.

6 Gao HY, Deng SB, Obach RS. A Simple Liquid Chromatography-Tandem Mass Spectrometry Method to Determine Relative Plasma Exposures of Drug Metabolites across Species for Metabolite Safety Assessments. Drug Metabolism and Disposition. 2010;38(12):2147-56.
